# Biophysical Techniques for Detection of cAMP and cGMP in Living Cells

**DOI:** 10.3390/ijms14048025

**Published:** 2013-04-12

**Authors:** Julia U. Sprenger, Viacheslav O. Nikolaev

**Affiliations:** Emmy Noether Group of the DFG, Department of Cardiology and Pneumology, European Heart Research Insitute Göttingen, Georg August University Medical Center, University of Göttingen, Göttingen 37075, Germany; E-Mail: julia.sprenger@med.uni-goettingen.de

**Keywords:** cAMP, cGMP, Förster resonance energy transfer (FRET), bioluminescence resonance energy transfer (BRET), imaging, compartmentation, microdomain

## Abstract

Cyclic nucleotides cAMP and cGMP are ubiquitous second messengers which regulate myriads of functions in virtually all eukaryotic cells. Their intracellular effects are often mediated via discrete subcellular signaling microdomains. In this review, we will discuss state-of-the-art techniques to measure cAMP and cGMP in biological samples with a particular focus on live cell imaging approaches, which allow their detection with high temporal and spatial resolution in living cells and tissues. Finally, we will describe how these techniques can be applied to the analysis of second messenger dynamics in subcellular signaling microdomains.

## 1. Introduction

The cyclic nucleotides 3′,5′-cyclic adenosine monophosphate (cAMP) and 3′,5′-cyclic guanosine monophosphate (cGMP) are universal second messengers which regulate a plethora of cellular functions [1].

Stimulation of various G-protein coupled receptors (GPCRs) leads to either activation or inhibition of cAMP production via stimulatory (G_s_) or inhibitory (G_i_) G-proteins, respectively. These G-proteins modulate the activity of several families of the cAMP producing enzymes adenylyl cyclases (ACs), which convert adenosine triphosphate to cAMP. An increase in intracellular cAMP levels induces several downstream effects, which strongly depend on the cell type. For example, cAMP has been shown to be involved in memory formation [2,3], insulin secretion [4–6], gene expression and metabolism [7], regulation of heart rate [8], and immune reactions [9–11].

cAMP activates three different types of downstream effectors, in particular cyclic nucleotide gated channels (CNGCs) [12,13], exchange protein directly activated by cAMP (Epac) [14–16], and cAMP-dependent protein kinase or protein kinase A (PKA) [17,18]. PKA consists of two regulatory (R) and two catalytic (C) subunits, which build a tetramer. Upon cAMP binding, free C-subunits dissociate from R-subunit dimers [19,20]. The C-subunits then phosphorylate a myriad of proteins, such as cAMP response element-binding (CREB) protein, which serve as transcription factors [7,21]. In addition, PKA phosphorylates several other effector proteins such as L-type calcium channels [22], ryanodine receptor [23], troponin I (TnI) [24], and phospholamban (PLN) [25,26], which are involved in the regulation of calcium homeostasis and excitation/contraction coupling in muscle cells [27].

The cellular production of cGMP is mediated by two families of guanylyl cyclases (GCs): transmembrane particulate (pGC) and soluble guanylyl cyclases (sGCs), which convert guanosine triphosphate to cGMP. pGCs are activated by the natriuretic peptides (NP) such as atrial (ANP), brain (BNP), and C-type (CNP) [28,29], whereas the sGCs can be stimulated by nitric oxide (NO) [30]. cGMP activates the cGMP-dependent protein kinase or protein kinase G (PKG) which in turn phosphorylates several downstream targets responsible for diverse functions of cGMP such as regulation of vascular tone, gastrointestinal function, neuronal activity, and many others [31]. cGMP can also regulate cAMP levels by activating or inhibiting specific phosphodiesterases (PDEs), the enzymes responsible for cAMP and cGMP degradation [32,33].

Even within the same cell, these cyclic nucleotides trigger different responses depending on the extracellular stimulus and the stimulated receptor. What are the molecular mechanisms behind these diverse effects? Over the last few decades, evidence has been accumulating that cyclic nucleotide signaling is not uniformly distributed in the cytosol, but rather, is organized in subcellular signaling microdomains [34–38]. Each of these microdomains contains a specific subset of differentially localized GPCRs, protein kinases, specific PDEs, and A-kinase anchoring proteins (AKAPs). These AKAPs are responsible for targeting PKA, some other protein kinases, and phosphatases in close proximity to their substrates [39–42]. PDEs are cyclic nucleotide hydrolyzing enzymes that shape and maintain intracellular gradients of cAMP and cGMP and play also an important role for the cGMP/cAMP crosstalk [32,43,44]. The concept of local cAMP or cGMP signaling in the structurally defined microdomains is known as cyclic nucleotide compartmentation. This widely accepted paradigm emphasizes the need for cyclic nucleotide detection methods that can be used to detect subcellular fluctuations of cAMP and cGMP with high temporal and spatial resolution in real-time.

In this review, we will summarize state-of-the-art techniques to measure cyclic nucleotide concentrations in cells and tissues. In particular, we will focus on biophysical approaches to study cAMP and cGMP compartmentation and dynamics in living cells with high temporal and spatial resolution.

## 2. Techniques to Measure Cyclic Nucleotides

### 2.1. Biochemical Methods

Radioimmunoassays (RIAs) allow quantification of total cAMP or cGMP concentrations in various cells and tissues [45–47]. RIAs use immobilized anti-cAMP or anti-cGMP antibodies and ^125^I-labeled cAMP/cGMP as a tracer molecule. The ^125^I-labeled cAMP/cGMP binds to the specific antibodies, and a radioactive signal can be detected. Upon addition of a cell or tissue sample to the anti-cAMP/cGMP antibodies, the cAMP or cGMP from the sample competes with the ^125^I-labeled cAMP/cGMP and the radioactive signal decreases, inversely proportional to the cAMP/cGMP concentration in the sample. Using a calibration curve, the amount of free cAMP or cGMP in a sample can be determined.

There are also several non-radioactive approaches to measure cyclic nucleotide concentration *in vitro*, such as enzyme-linked immunoassays. Here, the cyclic nucleotide from the sample binds to a specific first antibody and competes with the cGMP conjugated to alkaline phosphatase. The reagent mixtures are normally incubated in a secondary antibody coated multiwell plate, and the enzyme activity can be quantified by adding a substrate, which is converted into a colored product. The different antibody-based techniques are described in more detail elsewhere [47]. In principle, it is also possible to use Western blot analysis as an indirect way to detect changes in cyclic nucleotide levels. cAMP and cGMP activate PKA and PKG, respectively, and these kinases, in turn, phosphorylate several substrates. The phosphorylation status of these target proteins can be detected by phospho-specific antibodies as an indirect measure of intracellular cAMP and cGMP concentrations.

Despite being quite sensitive and specific, all biochemical methods described above require high amounts of cells or tissues that need to be disrupted. This makes it impossible to analyze real-time dynamics of cyclic nucleotides in various subcellular microdomains under physiologically relevant conditions. Therefore, it is necessary to use additional approaches which allow the visualization of cyclic nucleotide fluctuations in single living cells with high temporal and spatial resolution as described in the following sections.

### 2.2. Cyclic Nucleotide Gated Channels (CNGCs)

**Figure 1:**
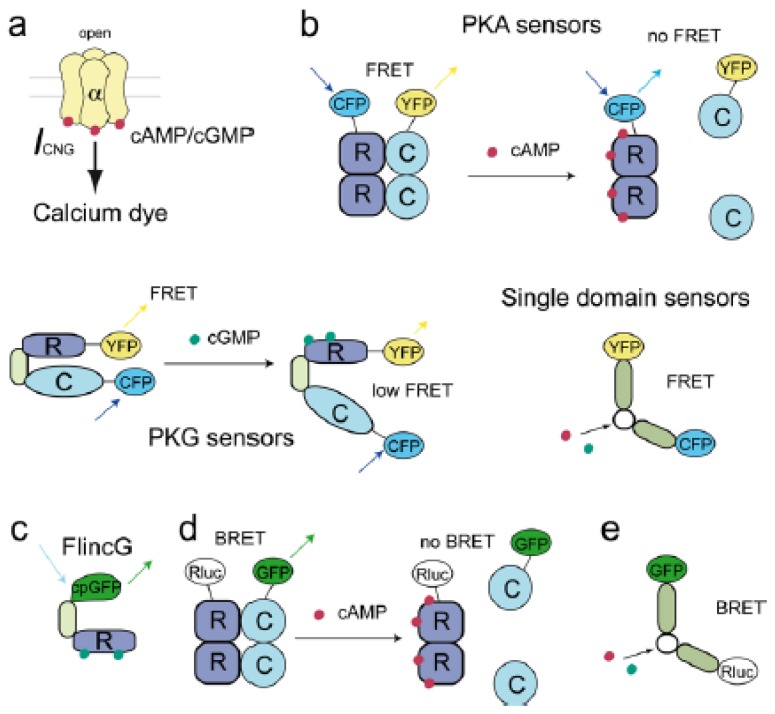
Common principles of biosensors designed to measure cAMP and cGMP. (**a**) Cyclic nucleotides can be monitored by directly measuring cyclic nucleotide gated channel (CNGC) currents or calcium influx through these channels by a calcium sensitive dye; (**b**) FRET sensors can be constructed based on the fluorophore-labeled protein kinase A (PKA) heterotetramer (R and C subunits), partially truncated protein kinase G (PKG) or single cAMP or cGMP binding domains; (**c**) Single circularly permuted cpGFP fused to cGMP binding sites from PKG can be used as a sensor for cGMP termed FlincG; (**d**,**e**) BRET biosensors use similar parts of proteins fused to Rluc and GFP.

Cyclic nucleotide gated channels (CNGCs) are nonselective cation channels in the plasma membrane, which consist of four subunits, each bearing an intracellular binding site for cAMP and cGMP. The channels are activated upon binding of these cyclic nucleotides [13,48,49] leading to a measurable cation current and an increase in intracellular calcium [50]. This CNG current (I_CNG_) can be recorded by a patch-clamp technique. Alternatively, calcium influx through these channels can be visualized using calcium sensitive dyes [51–53]. Both approaches prove an indirect measure of subsarcolemmal cyclic nucleotide concentrations (see Figure 1a).

Rich and colleagues introduced rat olfactory CNGC α-subunits as sensors for subsarcolemmal cAMP (see Table 1). These sensors can be expressed in various cell types using transfection or adenoviral gene transfer. When heterologously expressed, the α-subunits form homomultimeric channels. In C6-2B glioma cells, stimulation of endogenous ACs with the direct activator forskolin resulted in high cAMP concentrations close to the channels, while the global cytosolic cAMP concentration remained relatively low [54]. This led to the conclusion that CNGCs co-localize with ACs in the subsarcolemmal compartment, supporting the theory of compartmentalized cAMP signaling.

Wildtype CNGC α-subunits have a relatively low affinity for cAMP (~36 μM), while showing a much higher affinity for cGMP (~1.6 μM). Therefore, a series of mutants with increased affinity for cAMP (up to 1 μM in the C460W/E583M double-mutant) were generated and used to measure compartmentalized cAMP signaling in various cells, including adult rat ventricular cardiomyocytes (ARVMs) [53,55]. Due to their high affinity for cGMP, wildtype CNGCs have been used for real-time monitoring of cGMP. This has been extensively done to understand the visual transduction in retinal rods which show endogenous CNGC expression [56]. It was possible to unravel the interaction of cGMP specific PDEs and GCs [57], to understand the nature of single photon response [58] and to determine the cGMP concentration required for the dark current activation [59]. In ARVMs, adenoviral expression and measurements using wildtype CNGCs uncovered the role of various PDE families in shaping subcellular cGMP dynamics [60] and the regulatory role of PKG in cGMP accumulation in ARVMs [61] (see Section 3).

Although CNGCs as biosensors helped clarify several molecular mechanisms involved in the regulation of compartmentalized cyclic nucleotide signaling at the membrane, this technique has many important limitations, such as, its restriction to only one subsarcolemmal compartment and low cAMP/cGMP selectivity. The need for rather sophisticated electrophysiological measurements has further restricted the broad application of the technique.

### 2.3. Förster Resonance Energy Transfer (FRET) Based Sensors

The techniques described above are suitable approaches to measure cAMP and cGMP concentrations in cell lysates or to monitor their real-time dynamics close to the plasma membrane. However, these techniques either lack any spatial resolution or are too restricted to a specific location within the cell. Therefore, other methods have been developed to monitor cyclic nucleotide dynamics in living cells under real-time conditions. One important approach uses Förster resonance energy transfer (FRET) to generate biosensors for cyclic nucleotides. FRET is a non-radiative energy transfer between an excited donor and an acceptor fluorophore which leads to specific fluorescence emission of the acceptor without its direct excitation [62]. The donor and the acceptor fluorophores need to be in close proximity, usually at a distance of less than 10 nm, and in favorable spatial orientation to allow the energy transfer to take place [63]. The degree of FRET can be measured by various approaches, of which the most popular is represented by simple ratiometry. In this case, emission intensities of the donor and the acceptor molecules upon donor excitation are detected, and the FRET signal is calculated on a donor/acceptor or acceptor/donor ratio. Based on FRET, several biosensors for cAMP and cGMP have been developed which follow some common designs (see Figure 1b, Table 1).

#### 2.3.1. FRET Sensors to Detect cAMP

##### 2.3.1.1. Protein Kinase A (PKA) Based cAMP Sensors

The first approach to measure intracellular cAMP signals by the FRET technique was based on PKA. Adams and coworkers chemically labeled catalytic (C) or regulatory (R) subunits of the PKA with organic fluorophores fluorescein (donor) and rhodamine (acceptor) to allow energy transfer between these fluorophores in the PKA holoenzyme complex R_2_C_2_. Because PKA dissociates upon cAMP binding, the FRET signal decreases due to an intracellular cAMP increase. This FRET probe is called FlCRhR (fluorescein-labeled PKA catalytic subunit and a rhodamine-labeled regulatory subunit, pronounced “flicker”) [64].

FlCRhR was used in several studies to understand cAMP dynamics in neuronal networks, for example in *Aplysia* sensory neurons and the lobster stromatogastric ganglion, and to analyze the interactions of cAMP and Ca^2+^ oscillations in embryonic spinal neurons [65–67]. In addition, FlCRhR was used to investigate cAMP dynamics in mouse and hydrozoan oocytes [68,69] and, in combination with the patch clamp method, in isolated frog ventricular myocytes [70]. Production of chemically labeled proteins such as the FlCRhR probe, its purification and subsequent microinjection into living cells using micromolar concentrations is very complex and makes it desirable to develop more feasible, genetically encoded FRET sensors.

Manuela Zaccolo *et al.* were the first to develop a genetically encoded cAMP FRET probe using PKA and various green fluorescent protein (GFP) mutants [71]. First, they fused blue fluorescent proteins BFP and GFP to the PKA RII and C subunits, respectively, and were able to measure real-time cAMP dynamics in various cell types after co-transfection of cells with the two plasmids encoding for the labeled subunits [72]. Since, especially, the BFP is prone to photobleaching, they later switched to another, more suitable FRET pair, the enhanced cyan (CFP, fused to the RII subunit) and the enhanced yellow (YFP, fused to the C subunit) fluorescent proteins [73]. In neonatal rat ventricular cardiomyocytes (NRVMs), this sensor showed a striated pattern due to interaction with AKAPs close to the Z-lines and allowed the first cAMP measurements in cAMP microdomains. Using these sensors, it was possible to show the importance of specific PDE isoforms, in particular PDE3 and PDE4, for cAMP compartmentation in NRVMs [74]. In addition, adenoviral gene transfer was used to express the PKA based sensor in adult cardiomyocytes [75,76]. Further microdomain-specific FRET sensors based on PKA have been developed (see Section 3). Despite their great contribution to understanding cAMP signaling in living cells, PKA based FRET sensors have several disadvantages, in particular, equal expression of both sensor subunits to form a functional heterotetramer is necessary. There also might be a possible interaction of the sensor subunits with endogenous wildtype R or C subunits, and the cooperative cAMP binding to PKA subunits leads to relatively slow sensor kinetics [77,78]. Therefore, simpler single-chain PKA based FRET sensors without catalytic properties and faster kinetics were developed, e.g., PKA-camps which contains a single cAMP binding domain of the PKA RIIα subunit (PKA-cAMP sensor) [78].

In addition to measuring cAMP levels directly by FRET-based cAMP biosensors, the dynamics of its signaling can be further investigated by looking at the catalytic activity of the PKA using the so-called A-kinase activity reporter (AKAR). This is a family of FRET-based sensors (AKAR1-3), which contain a PKA substrate sequence and a phosphate-binding acceptor domain sandwiched between eCFP and YFP/Venus [79–81]. These sensors are being widely applied to monitor PKA activity dynamics with high temporal and spatial resolution by FRET. Recently, an improved version of these reporters called AKAR4 has been published. This sensor uses cerulean instead of CFP as a donor fluorophore. AKAR4 has been fused to several lipid modification domains to target it to the plasma membrane and to measure compartmentalized PKA activity in this subcellular compartment [82].

##### 2.3.1.2. Epac-Based cAMP Sensors

Several Epac-based, single-chain FRET sensors were developed and published in 2004 [78,83,84]. Nikolaev and coworkers used single cAMP binding domains of either human Epac1 or murine Epac2 and fused them to CFP and YFP in different positions to generate Epac1-camps and Epac2-camps sensors, respectively [78]. cAMP binding induces a conformational shift within the sensors leading to a decrease of the FRET signal. Purified Epac2-camps showed significantly faster kinetics also at low cAMP concentrations when compared to the tetrameric PKA sensor described earlier [72]. In β_1_-adrenergic receptor (β_1_AR) expressing cells Epac1-camps, although having a slightly lower cAMP affinity than Epac2-camps (~2 μM *vs*. ~1 μM), showed larger FRET ratio changes, which is why Epac1-camps has been used in many other studies. Due to its uniform distribution in the cytosol of transfected cells, Epac1-camps could be used to detect cAMP signals rapidly diffusing (~40 μm/s) throughout neurons and macrophages upon receptor stimulation [78]. Later on, Epac2-camps was used in an adenovirus based approach to monitor PDE2 activity in primary cultured adrenal *zona glomerulosa* cells [85]. A transgenic reporter mouse line (CAG-Epac1-camps) was created with ubiquitous expression of the Epac1-camps sensor [86]. This allows the investigation of GPCR-cAMP signaling under even more physiological conditions in living tissues and single cells.

In parallel, Ponsioen *et al.* created a cAMP FRET probe using the full length or partially truncated Epac1 protein fused to CFP and YFP (CFP-Epac-YFP) [84]. This catalytically active biosensor was transfected and expressed in several mammalian cells where it localized in the cytosol and partially at the perinuclear envelope. Upon stimulation with cAMP elevating agents such as forskolin, the FRET signal from the sensor decreased due to conformational changes. The catalytically inactive mutant of the CFP-Epac-YFP mainly located to the cytosol, showed a higher FRET response than the primary sensor construct (about 30% total FRET change, affinity to cAMP of ~14 μM) and had an improved signal-to-noise ratio. The authors could also show an extended dynamic range of their single-chain Epac-based biosensors compared to the PKA probes [72].

Di Pilato and coworkers developed several FRET probes named ICUE (indicator of cAMP using Epac) using either the full length Epac1 or truncated versions of Epac2 sandwiched between CFP and Citrine [83]. Upon cAMP elevation in transfected mammalian cells, the FRET signal decreased, and the FRET sensor with the biggest FRET response, called ICUE1, was used for further experiments. ICUE1 showed a uniform distribution in the cytosol. In addition, several targeted versions were developed to measure local cAMP levels at the plasma membrane, in the nucleus or mitochondria [83]. Violin and coworkers developed an improved version of the ICUE1 cAMP biosensor, namely ICUE2, which showed larger FRET signals and minimally affected cellular functions [87].

All these single-chain Epac based FRET probes for cAMP do not show the technical problems associated with the tetrameric PKA sensors such as unequal subunit expression (see Table 1). Therefore, these sensors are an easy tool to measure cAMP dynamics in living cells.

##### 2.3.1.3. Cyclic Nucleotide Gated Channels (CNGC) Based cAMP Sensors

Cyclic nucleotide gated channels (CNGCs) contain a C-terminal cyclic nucleotide binding domain that is involved in channel gating [8]. Nikolaev *et al.* used a single cAMP binding domain from the murine hyperpolarization activated cyclic nucleotide-gated potassium channel 2 (HCN2) and sandwiched it between CFP and YFP [88]. The resultant cytosolic cAMP biosensor was called HCN2-camps and showed high dynamic range and optimized cAMP sensitivity (~ 6 μM) for cells with higher basal cAMP concentrations. This sensor was useful for real-time measurements of cAMP dynamics in cardiomyocytes. The authors generated a transgenic mouse line, which expresses HCN2-camps in a tissue-specific manner and measured cAMP responses in single freshly isolated adult cardiomyocytes. These experiments showed that local stimulation of β_1_-AR led to far-reaching cAMP diffusion patterns across the entire cell, while β_2_-AR-cAMP signals were highly locally confined by an unknown mechanism [88]. This approach allowed direct visualization of different cAMP spatial patterns induced by two G_s_-coupled receptors.

#### 2.3.2. FRET Sensors to Detect cGMP

In addition to electrophysiological recordings using CNGCs as subsarcolemmal cGMP sensors, several genetically encoded FRET-based cGMP biosensors were developed [89] (see Figure 1b). Many of these sensors are based on partially truncated PKG containing both cGMP binding domains, sandwiched between CFP and YFP [90–92]. Honda and coworkers used N- or C-terminally truncated PKG-Iα and fused it to CFP and YFP or Citrine. One N-terminally truncated sensor version showed a decreased FRET signal upon cGMP binding and was called Cygnet-1 (cyclic GMP indicator using energy transfer). In contrast, the slightly longer CGY cGMP biosensors by Sato *et al.* showed an increase of the FRET signal upon cGMP elevation [92]. To delete the kinase activity of Cygnet-1, a catalytically inactive mutant Cygnet-2 was generated [90]. Thanks to the Cygnet biosensors it was possible to gain novel insights into the regulation of cGMP dynamics in several cell types [44,93–97]. The dynamic range and the temporal resolution of these long PKG-based biosensors is relatively low which led to the development of shorter cGMP sensors containing only single cGMP binding domains [91,98].

Using the single cGMP binding domain B from PKG-I, or the GAF domains from PDE2 and PDE5 fused to CFP and YFP, Nikolaev *et al.* generated several fluorescent cGMP sensors. Upon cGMP increase, PKG-I based sensors showed a decrease in the FRET signal, whereas the GAF based probes responded with a FRET increase ~2-fold greater than those of the CGY and Cygnet constructs [98]. The most promising sensor generated in this study was cGES-DE5 (cGMP energy transfer sensor derived from PDE5), which showed the highest cGMP to cAMP selectivity, rapid responses to intracellular cGMP signals and good FRET signal amplitudes. This makes the sensor a suitable tool to measure cGMP dynamics with high temporal and spatial resolution in real-time. However, the sensitivity to cGMP was still quite low. By exchanging YFP and CFP in cGES-DE5 with green (T-Sapphire) and red (Dimer2) fluorescent proteins, respectively, the cGMP affinity of the new sensor redcGES-DE5 could be, unexpectedly, further increased about 40-fold, making it a promising tool for the detection of low cGMP concentrations [99].

Russwurm and coworkers used tandem cGMP binding domains from PKG and GAF domains from PDE5 fused to CFP and YFP in a systematic approach to generate several new cGMP-FRET probes [91]. Three cGMP indicators (cGi-500, −3000, −6000) with different EC_50_ for cGMP based on tandem cGMP binding domains of PKG were selected. All FRET probes showed the cGMP affinities of 500, 3000, and 6000 nM, respectively, high cGMP over cAMP selectivity, fast kinetics, and a greater dynamic range than the cGMP biosensors described above [91].

When using cAMP and cGMP FRET biosensors it is important to confirm the specificity of the signal and the absence of artifacts, for example, related to changes of pH, bivalent ions, or ATP [100–102]. In addition, it is useful to calibrate the sensors and to convert FRET ratios into absolute cAMP and cGMP concentrations, which can be done according to previously established protocols [103,104].

### 2.4. Single GFP-Linked Biosensors

cGMP signaling is believed to act in spatially defined compartments at low concentrations [37,60,105,106], which makes it necessary to create highly sensitive cGMP sensors which allow high temporal and spatial resolution of intracellular cGMP dynamics. Nausch *et al.* developed non-FRET cGMP biosensors which were called fluorescent indicators of cGMP (FlincGs), containing two in-tandem PKG-I derived cGMP binding sites fused to the N-terminus of a circularly permuted (*i.e.*, having a changed order of amino acid sequence) enhanced GFP (cpGFP) [107,108] (see Figure 1c). They used the differently truncated PKG-I variants α, β, δ, which differ in their N-terminal regulatory domain leading to α-FlincG, β-FlincG, and δ-FlincG, respectively. These sensors show diverse dynamic ranges, dissociation constants and selectivities for cGMP. Binding of cGMP to the PKG-derived sequence leads to a conformational changes and an increase of fluorescence from cpGFP. Especially rapid dissociation and association kinetics of δ-FlincG (affinity of 170 nM for cGMP and 48 μM for cAMP) allow detection of rapid changes in cGMP concentrations using confocal microscopy. The δ-FlincG biosensor was expressed in rat vascular smooth muscle cells (VSMCs) via adenoviral gene transfer. The cells were treated either with the NO-donor DEA-NO to activate sGC or with ANP to activate its receptor GC-A. Confocal microscopy was used to analyze spatially resolved changes of cGMP in real time. Stimulation with DEA-NO led to a transient and global increase of cGMP concentration, while ANP produced a sustained but clearly submembrane cGMP signal. ANP stimulation upon PDE5 inhibition had the same effect as DEA-NO stimulation leading to the conclusion that in rat VSMCs, sGC and pGC create distinct intracellular cGMP patterns, which are controlled by PDE5 [108].

δ-FlincG was also used to analyze cGMP signaling in the plant *Arabidopsis thaliana.* Isner and Maathuis investigated the influence of NO, gibberellic acid [109] and other plant hormones such as abscisin acid and brassinosteroids [110] on changes in cGMP concentrations, showing the broad application potential of this newly developed cGMP biosensor.

### 2.5. Bioluminescence Resonance Energy Transfer (BRET) Based Sensors

The previously described FRET technique allows real-time measurements of cyclic nucleotide dynamics in intact cells. Some cells might show a relatively high level of autofluorescence upon fluorophore excitation which limits FRET-based high-throughput screenings approaches [113].

Bioluminescence resonance energy transfer (BRET) is a non-radiative energy transfer between a donor enzyme after oxidation of its luminescent substrate and an acceptor fluorophore. The lack of an external light source makes this approach suitable for the investigation of light-sensitive systems such as plant cells or photoreceptors [114]. The donor enzyme usually is a variant of the *Renilla reniformis* luciferase (Rluc), and coelenterazine is typically used as a substrate. The acceptor fluorophores are usually represented by various GFP variants [115]. Similar to the FRET technique, donor and acceptor molecules need to get into close proximity, less than 10 nm [63], to enable energy transfer and the detection of the fluorescent signal. The donor and the acceptor emissions are detected and the BRET signal is calculated from their ratio [63,114].

Prinz and coworkers introduced the first BRET cAMP biosensor based on PKA [116]. They fused Rluc to the regulatory RI and RII subunit, creating two different donor proteins for the BRET system, RI-Rluc and RII-Rluc, respectively. The acceptor protein is composed of the catalytic subunit fused to a GFP variant (GFP-C). COS7 cells were either co-transfected with RI-Rluc and GFP-C or with RII-Luc and GFP-C to investigate PKA subunit interactions in living cells and to compare PKA-I and PKA-II signals under real-time conditions. Because PKA regulatory and catalytic subunits dissociate upon cAMP binding, the BRET signal decreases in a dose-dependent manner with increasing intracellular cAMP concentrations. Upon β_2_-AR stimulation with isoproterenol, only the RII-Luc/GFP-C system showed a decreased BRET signal, indicating a close proximity of the PKA-RII isoform to the β_2_-AR. However, an increase of cAMP due to the treatment of the cells with isoproterenol and PDE inhibitors could be detected by both PKA isoforms [116].

In addition to this two-protein BRET system, two different intramolecular BRET sensors based on the Epac protein were created [117,118]. Jiang and coworkers used a cytosolic mutant form of the human Epac1 protein and fused it to Rluc and the YFP variant Citrine as the BRET pair. The sensor showed a strong BRET signal under basal conditions, which could be even further improved by using a circularly permuted version of Citrine in the sensor named CAMYEL (cAMP sensor using YFP-Epac-RLuc). They used this improved sensor for rapid and quantitative monitoring of intracellular cAMP concentrations in mouse macrophage-like RAW264.7 cells where an increase of cAMP concentration leads to a decrease of the BRET signal. They showed that sphingosine-1-phosphate (S1P) might amplify cAMP signals induced by GPCR ligands (such as isoproterenol or prostaglandin 2) via binding to S1P_2_ receptors and activating G_13_ proteins [118].

Membrane targeted variants of CAMYEL were used in HEK293 cells to compare its BRET signals with those reported by the cytosolic CAMYEL. These experiments demonstrated differential PDE distribution between the two compartments of these cells [119].

The second Epac-based BRET sensor for cAMP detection was created by Barak *et al*. [117] who fused the Rluc and Citrine to the cAMP binding domain from Epac. They expressed the sensor in HEK293 cells and evaluated the activity of the potential trace-amine associated receptor 1 ligands in a cell-based screening assay [117].

To monitor cGMP signals in HEK293T cell lysates, Biswas and coworkers generated cGMP specific BRET sensors based on the GAF domain from PDE5 [120]. They sandwiched either a wildtype or a mutant variant of the GAF domain between GFP and Rluc, similar to the design of the previously described cGES-DE5 FRET sensor. The mutant form was not able to bind cGMP and served as a control for the BRET specificity in this study. The authors could show that GAF domains may act as intracellular cGMP sinks and that, under basal conditions, there might be a cGMP reservoir in cells expressing GAF domain containing proteins. This cGMP-BRET sensor may be further used for high-throughput screening to identify new activators or inhibitors which facilitate or prevent cGMP binding to the GAF domain of PDE5 [120], similar to what has been started with the FRET-based sensors [121].

## 3. Analysis of Compartmentalized Cyclic Nucleotide Signaling

As described above, cyclic nucleotides trigger several intracellular effects depending on the specific extracellular stimulus. This led to a widely accepted view that cyclic nucleotides act in spatially defined subcellular compartments [37]. The first evidence for cAMP acting in a compartmentalized fashion was provided over 30 years ago. The β-adrenergic agonist isoproterenol and the prostaglandin receptor agonist prostaglandin E1 (PGE1) both led to cAMP increase in cardiac tissue, but only isoproterenol was able to induce changes in TnI phosphorylation [34,122]. Accordingly, isolated membrane and cytosolic fractions from adult rabbit cardiomyocytes showed different cAMP and PKA activities upon isoproterenol or PGE1 stimulation. In particular, isoproterenol was able to stimulate cAMP in both fractions, whereas PGE1 did so exclusively in the cytosolic fraction [35,36].

Inspired by these findings, several real-time imaging techniques described above have been further developed to monitor cyclic nucleotide compartmentation in different cell types. Jurevicius and colleagues were the first to show the connection between local pools of cAMP and L-type Ca^2+^ channel current (I_Ca_) in single living cells [123]. They used isolated frog cardiomyocytes and treated them with isoproterenol and the direct AC activator forskolin in a special chamber designed for simultaneous whole-cell patch-clamp recordings of I_Ca_ using a double-barreled micropipette. I_Ca_ signals, as an indirect reporter of cAMP/PKA activity, revealed that β_2_AR stimulation leads to spatially confined cAMP signals as long as PDEs are active. On the other hand, direct activation of ACs induced a global intracellular cAMP increase, supporting the concept of cAMP compartmentation [123].

As described above, CNGCs are valuable tools to measure cAMP and cGMP signals in the subsarcolemmal compartment via I_CNG_ current or calcium imaging (see 2.2). Rochais and colleagues pioneered measurements of compartmentalized cAMP using CNGCs heterologously expressed in single living ARVMs [53]. This approach has uncovered the role of various PDE isoforms in confinement of cAMP and cGMP signals in ARVMs. In particular, PDE3 and PDE4 together were found to control cAMP generated by selective _2_AR stimulation [124]. The group of Thomas Rich could detect compartmentalized cGMP signaling upon either pGC or sGC stimulation in VSMCs [105]. In ARVMs, PDE2 has been shown to control the cGMP pools produced by the ANP and BNP receptor GC-A at the plasma membrane, while both PDE2 and PDE5 in concert were responsible for cGMP compartmentation around sGC [60]. Local cGMP pools were also shown to stimulate cGMP degradation via PKG-dependent phosphorylation of PDE5 and to increase the GC-A activity by a PKG-dependent feed-forward mechanism [61].

FRET technology has been used to gain insights into cAMP and cGMP compartmentation in various cell types by developing several targeted FRET sensors (see Table 2). Using the PKA based biosensors (see 2.3.1.1) which are localized to specific subcellular sites due to their interaction with AKAP, it has been shown that PDEs might act as cAMP sinks in HEK293 leading to several independent subcellular compartments with different cAMP concentrations [125]. Recently, evidence has been provided that RI and RII isoforms of the PKA, due to selective AKAP binding, can define independent subcellular compartments associated with specific PDE subsets for targeted cAMP degradation. To directly monitor cAMP levels in the distinct compartments around PKAI and PKAII isoforms, Epac1-camps has been fused to the N-terminal dimerization-docking domains of RI and RII subunits to generate RI_epac and RII_epac sensors. βAR stimulation by catecholamines induced cAMP increases exclusively in the PKA-RII microdomain, leading to PLN and TnI phosphorylation, whereas PGE1 stimulation leads to PKA-RI activation and phosphorylation of yet unknown local substrates [126]. Using PKA-RI and PKA-RII based FRET sensors, Stangherlin and colleagues uncovered opposing microdomain-specific effects of cGMP pools produced by GC-A and sGC on cAMP concentrations in the different compartments via specific PDEs such as PDE2 and PDE3 [44].

PDEs are major regulators of cyclic nucleotide gradients. To directly monitor cAMP and cGMP dynamics in the direct vicinity of various PDEs, Herget and colleagues fused Epac1-camps or cGES-DE2 to the N-terminus of PDE3, PDE4 or PDE5 [127]. These sensors can be also used to study the regulation of PDE activity by PKA or PKG.

Epac1-camps has also been fused to the small heat shock protein 20 (Hsp20). This newly sensor construct uncovered a direct interaction and sequestration of PDE4 to Hsp20 identifying a novel functionally relevant Hsp20:PDE4 complex in NRVMs [128].

Targeting of the PKA activity reporter AKAR3 [79] to the transmembrane domain of PLN led to a sarcoplasmic reticulum (SR) targeted biosensor named SR-AKAR3 [129]. This sensor is suitable for analysis of PKA activity dynamics at the cytosolic side of the cardiomyocyte SR membrane where it revealed a high basal PDE4 activity which, via cAMP, controls local pools of PKA [129].

To further increase spatial resolution and more precisely study subcellular microdomains, FRET can be combined with non-optical imaging techniques such as scanning ion conductance microscopy (SICM). The group of Julia Gorelik has developed a SICM/FRET hybrid method, which allows localization of active GPCRs and monitoring of compartmentalized cAMP with nanometer precision in living cells [132]. This technique showed that in healthy cardiomyocytes, β_2_AR is localized exclusively in the T-tubules, whereas β_1_AR was detected in both T-tubular and non-tubular membrane areas. In chronic heart failure models, β_2_ARs redistribute from T-tubules to detubulated membrane, which leads to changed cAMP compartmentation, in particular to the far-reaching cAMP signals from this receptor [132]. The combination of SICM and FRET measurements is a promising approach to analyze the localization of GPCRs and their interactions with subcellular cAMP and cGMP microdomains.

Dyachok and colleagues targeted a truncated PKA RII-subunit labeled with CFP to the membrane of pancreatic β-cells. Due to the dynamic interaction with a co-expressed PKA C-subunit fused to YFP it was possible to measure cAMP oscillations underneath the plasma membrane [130]. Redistribution of activated C-YFP to the cytosol after cAMP-induced holoenzyme dissociation was monitored by total internal reflection fluorescence microscopy [133] and served as an indicator for increased subsarcolemmal cAMP concentrations. In β-cells stimulated with a glucagon-like peptide 1 or glucose, cAMP levels were oscillating which might be responsible to a pulsatile insulin release [130,134]. Similar to this approach, Epac2-camps has been fused on its N-terminus to a 10 amino acid sequence from Lyn kinase or to the full length AC8 sequence [111]. This approach allowed monitoring cAMP dynamics in various subsarcolemmal compartments and to detect discrete cAMP pools associated with ACs.

## 4. Conclusions and Outlook

Over the last few decades, a variety of powerful biophysical techniques and biosensors have been developed to allow direct visualization of cyclic nucleotides in intact living cells and tissues. These innovative methods should be further developed and applied to the analysis of compartmentalized cAMP and cGMP signaling. Compartmentation of cyclic nucleotides is a challenging field to investigate, and it will take a lot of effort to fully understand the complex interactions of receptors, PDEs, AKAPs, and cyclic nucleotides. Especially, imaging of cGMP is sometimes difficult because of its relatively low, compared to cAMP, concentrations. Therefore, new techniques and biosensors, which would provide high sensitivity, temporal and spatial resolution still need to be developed. Transgenic mouse models expressing genetically encoded cAMP and cGMP biosensors, either globally or targeted to specific microdomains, could greatly facilitate the investigation of cyclic nucleotide dynamics directly in functionally relevant subcellular compartments. Combination of such biosensors with the SICM technique could uncover the localization of the involved receptors and their interactions with cAMP and cGMP microdomains under normal and pathological conditions. Last but not least, the results gained in animal or cell culture models need to be verified in regard to their relevance for human cells and tissues.

## Figures and Tables

**Table 1:** Currently available Förster Resonance Energy Transfer (FRET) biosensors for cAMP, cGMP, and PKA activity.

Biosensor	Sensitivity	Advantages/Disadvantages	References
CNGC -subunit wildtype C460W/E583M mutant	cAMP EC_50_ = 36 μM cGMP EC_50_ = 1.6 μM cAMP EC_50_ = 1 μM	Low cAMP/cGMP selectivity. Restriction to the subsarcolemmal compartment	[53,55]
*FRET based biosensors*:			
FlCRhR (PKA based)	cAMP EC_50_ = 90 nM	Chemical labeling, purification and microinjection Relatively slow kinetics	[64]
R-CFP, C-YFP (PKA based)	cAMP EC_50_ = 0.5–0.9 μM	Multimeric. Here and below: genetically encoded	[72–74]
PKA-camps (PKA based)	cAMP EC_50_ = 1.9 μM	Single-chain architecture	[78]
AKAR1-3	Not applicable	Measures PKA catalytic activity in real time	[79–81]
AKAR4	Not applicable	Improved dynamic range	[82]
Epac1/2-camps (Epac based)	cAMP EC_50_ = 2.4/0.9 μM	Single-chain. Faster kinetics than for multimeric sensors	[78]
Epac2-camp300 CFP-Epac-YFP ( DEP,CD)	cAMP EC_50_ = 300 nM cAMP EC_50_ ~ 50 μM cAMP EC_50_ ~ 15 μM	High sensitivity Single-chain. Relatively low sensitivity	[104] [84,111,112]
ICUE1/2 (Epac based)	cAMP EC_50_ ~ 10–50 μM	As above	[83,87]
HCN2-camps (CNGC based)	cAMP EC_50_ = 6 μM	Good for cells with high basal cAMP concentrations	[88]
CGY-Del1	cGMP EC_50_ = 20 nM	Low cGMP/cAMP selectivity	[92,98]
Cygnet-1/2 (PKG based)	cGMP EC_50_ = 1.5/1.9 μM	Single-chain. Relatively low sensitivity and temporal resolution	[90]
cGES-DE2/5 (PDE2/5 based)	cGMP EC_50_ = 0.9/1.5 μM	Small size. Relatively low sensitivity	[98]
redcGES-DE5 (PDE5 based)	cGMP EC_50_ = 40 nM	High sensitivity	[99]
cGi-500/3000/6000 (PKG based)	cGMP EC_50_ = 500/3000/6000 nM	Small size. Relatively high sensitivity and dynamic range. Fast kinetics	[91]
*Non-FRET sensors*: FlincGs cGMP	EC_50_ = 150nM (δ-FlincG)	Good dynamic range. Rapid kinetics	[108]

**Table 2:** Currently available targeted FRET biosensors for cAMP, cGMP and PKA activity.

Targeted biosensor	Structure	Microdomain	References
RI_epac and RII_epac	N-terminal dimerization-docking domains of RI or RII fused to Epac1-camps	PKA-RI and PKA-RII	[126]
Epac1-camps-PDE3/4	Fusion of Epac1-camps to N-terminus of PDEs	PDE3/4	[127]
cGES-DE2-PDE5	Fusion of cGES-DE2 to N-terminus of PDE5	PDE5	[127]
Epac1-camps-Hsp20	Fusion of Epac1-camps to Hsp20	Hsp20	[128]
SR-AKAR3	Fusion of AKAR3 to the N-terminal helical transmembrane domain of phospholamban	SR membrane	[129]
pm PKA-RII-CFP/C-YFP	26 amino acid CAAX box sequence fused to the C-terminus of PKA-CFP	Subsarcolemmal	[130]
pmEpac2-camps and AC8-Epac2-camps pmEpac1-camps	10 amino acid sequence form Lyn kinase or AC8 are fused to N-terminus of Epac1/2-camps	Subsarcolemmal caveolar or associated with AC8	[111,112]
pm ICUE, NLS-ICUE,	mitoICUE Fusions of ICUE to CAAX box, nuclear localization signals or two different mitochondrial sequences	Subsarcolemmal, nuclear, mitochondrial	[83,131]

## References

[1] Beavo J.A., Brunton L.L. (2002). Cyclic nucleotide research -- still expanding after half a century. Nat. Rev. Mol. Cell Biol.

[2] Kandel E.R. (2001). The molecular biology of memory storage: A dialogue between genes and synapses. Science.

[3] Morozov A., Muzzio I.A., Bourtchouladze R., Van-Strien N., Lapidus K., Yin D., Winder D.G., Adams J.P., Sweatt J.D., Kandel E.R. (2003). Rap1 couples cAMP signaling to a distinct pool of p42/44MAPK regulating excitability, synaptic plasticity, learning, and memory. Neuron.

[4] Holz G.G. (2004). Epac: A new cAMP-binding protein in support of glucagon-like peptide-1 receptor-mediated signal transduction in the pancreatic beta-cell. Diabetes.

[5] Tengholm A., Gylfe E. (2009). Oscillatory control of insulin secretion. Mol. Cell. Endocrinol.

[6] Leech C.A., Chepurny O.G., Holz G.G. (2010). Epac2-dependent rap1 activation and the control of islet insulin secretion by glucagon-like peptide-1. Vitam. Horm.

[7] Altarejos J.Y., Montminy M. (2011). CREB and the CRTC co-activators: Sensors for hormonal and metabolic signals. Nat. Rev. Mol. Cell Biol.

[8] Zagotta W.N., Olivier N.B., Black K.D., Young E.C., Olson R., Gouaux E. (2003). Structural basis for modulation and agonist specificity of HCN pacemaker channels. Nature.

[9] Brudvik K.W., Tasken K. (2010). Modulation of T cell immune functions by the prostaglandin E(2) -cAMP pathway in chronic inflammatory states. Br. J. Pharmacol.

[10] Bodor J., Bopp T., Vaeth M., Klein M., Serfling E., Hunig T., Becker C., Schild H., Schmitt E. (2012). Cyclic AMP underpins suppression by regulatory T cells. Eur. J. Immunol.

[11] Torgersen K.M., Vang T., Abrahamsen H., Yaqub S., Tasken K. (2002). Molecular mechanisms for protein kinase A-mediated modulation of immune function. Cell. Signal.

[12] Biel M., Wahl-Schott C., Michalakis S., Zong X. (2009). Hyperpolarization-activated cation channels: from genes to function. Physiol. Rev.

[13] Craven K.B., Zagotta W.N. (2006). CNG and HCN channels: Two peas, one pod. Annu. Rev. Physiol.

[14] De Rooij J., Zwartkruis F.J., Verheijen M.H., Cool R.H., Nijman S.M., Wittinghofer A., Bos J.L. (1998). Epac is a Rap1 guanine-nucleotide-exchange factor directly activated by cyclic AMP. Nature.

[15] Kawasaki H., Springett G.M., Mochizuki N., Toki S., Nakaya M., Matsuda M., Housman D.E., Graybiel A.M. (1998). A family of cAMP-binding proteins that directly activate Rap1. Science.

[16] Gloerich M., Bos J.L. (2010). Epac: defining a new mechanism for cAMP action. Annu. Rev. Pharmacol. Toxicol.

[17] Tasken K., Aandahl E.M. (2004). Localized effects of cAMP mediated by distinct routes of protein kinase A. Physiol. Rev.

[18] Taylor S.S., Buechler J.A., Yonemoto W. (1990). cAMP-dependent protein kinase: framework for a diverse family of regulatory enzymes. Annu. Rev. Biochem.

[19] Krebs E.G., Beavo J.A. (1979). Phosphorylation-dephosphorylation of enzymes. Annu. Rev. Biochem.

[20] Taylor S.S., Kim C., Cheng C.Y., Brown S.H., Wu J., Kannan N. (2008). Signaling through cAMP and cAMP-dependent protein kinase: diverse strategies for drug design. Biochim. Biophys. Acta.

[21] Muller F.U., Boknik P., Knapp J., Linck B., Luss H., Neumann J., Schmitz W. (2001). Activation and inactivation of cAMP-response element-mediated gene transcription in cardiac myocytes. Cardiovasc. Res.

[22] Keef K.D., Hume J.R., Zhong J. (2001). Regulation of cardiac and smooth muscle Ca(2+) channels (Ca(V)1.2a,b) by protein kinases. Am. J. Physiol. Cell. Physiol.

[23] Takasago T., Imagawa T., Shigekawa M. (1989). Phosphorylation of the cardiac ryanodine receptor by cAMP-dependent protein kinase. J. Biochem.

[24] Bers D.M. (2002). Cardiac excitation-contraction coupling. Nature.

[25] Kirchberger M.A., Tada M., Repke D.I., Katz A.M. (1972). Cyclic adenosine 3′,5′-monophosphate-dependent protein kinase stimulation of calcium uptake by canine cardiac microsomes. J. Mol. Cell. Cardiol.

[26] MacLennan D.H., Kranias E.G. (2003). Phospholamban: a crucial regulator of cardiac contractility. Nat. Rev. Mol. Cell. Biol.

[27] Lompre A.M., Hajjar R.J., Harding S.E., Kranias E.G., Lohse M.J., Marks A.R. (2010). Ca2+ cycling and new therapeutic approaches for heart failure. Circulation.

[28] Kuhn M. (2003). Structure, regulation, and function of mammalian membrane guanylyl cyclase receptors, with a focus on guanylyl cyclase-A. Circ. Res.

[29] Potter L.R., Abbey-Hosch S., Dickey D.M. (2006). Natriuretic peptides, their receptors, and cyclic guanosine monophosphate-dependent signaling functions. Endocr. Rev.

[30] Pyriochou A., Papapetropoulos A. (2005). Soluble guanylyl cyclase: more secrets revealed. Cell Signal.

[31] Hofmann F., Feil R., Kleppisch T., Schlossmann J. (2006). Function of cGMP-dependent protein kinases as revealed by gene deletion. Physiol. Rev.

[32] Stangherlin A., Zaccolo M. (2012). cGMP-cAMP interplay in cardiac myocytes: A local affair with far-reaching consequences for heart function. Biochem. Soc. Trans.

[33] Zaccolo M., Movsesian M.A. (2007). cAMP and cGMP signaling cross-talk: role of phosphodiesterases and implications for cardiac pathophysiology. Circ. Res.

[34] Brunton L.L., Hayes J.S., Mayer S.E. (1979). Hormonally specific phosphorylation of cardiac troponin I and activation of glycogen phosphorylase. Nature.

[35] Brunton L.L., Hayes J.S., Mayer S.E. (1981). Functional compartmentation of cyclic AMP and protein kinase in heart. Adv. Cyclic Nucleotide Res.

[36] Buxton I.L., Brunton L.L. (1983). Compartments of cyclic AMP and protein kinase in mammalian cardiomyocytes. J. Biol. Chem.

[37] Fischmeister R., Castro L.R., Abi-Gerges A., Rochais F., Jurevicius J., Leroy J., Vandecasteele G. (2006). Compartmentation of cyclic nucleotide signaling in the heart: the role of cyclic nucleotide phosphodiesterases. Circ. Res.

[38] Zaccolo M. (2009). cAMP signal transduction in the heart: Understanding spatial control for the development of novel therapeutic strategies. Br. J. Pharmacol.

[39] Diviani D., Dodge-Kafka K.L., Li J., Kapiloff M.S. (2011). A-kinase anchoring proteins: scaffolding proteins in the heart. Am. J. Physiol. Heart Circ. Physiol.

[40] Mauban J.R., O’Donnell M., Warrier S., Manni S., Bond M. (2009). AKAP-scaffolding proteins and regulation of cardiac physiology. Physiology (Bethesda).

[41] Scott J.D., Dessauer C.W., Tasken K. (2013). Creating order from chaos: Cellular regulation by kinase anchoring. Annu. Rev. Pharmacol. Toxicol.

[42] Troger J., Moutty M.C., Skroblin P., Klussmann E. (2012). A-kinase anchoring proteins as potential drug targets. Br. J. Pharmacol.

[43] Rapundalo S.T., Solaro R.J., Kranias E.G. (1989). Inotropic responses to isoproterenol and phosphodiesterase inhibitors in intact guinea pig hearts: Comparison of cyclic AMP levels and phosphorylation of sarcoplasmic reticulum and myofibrillar proteins. Circ. Res.

[44] Stangherlin A., Gesellchen F., Zoccarato A., Terrin A., Fields L.A., Berrera M., Surdo N.C., Craig M.A., Smith G., Hamilton G. (2011). cGMP signals modulate cAMP levels in a compartment-specific manner to regulate catecholamine-dependent signaling in cardiac myocytes. Circ. Res.

[45] Brooker G., Harper J.F., Terasaki W.L., Moylan R.D. (1979). Radioimmunoassay of cyclic AMP and cyclic GMP. Adv. Cyclic Nucleotide Res.

[46] Harper J.F., Brooker G. (1975). Femtomole sensitive radioimmunoassay for cyclic AMP and cyclic GMP after 2′0 acetylation by acetic anhydride in aqueous solution. J. Cyclic Nucleotide Res.

[47] Williams C. (2004). cAMP detection methods in HTS: selecting the best from the rest. Nat. Rev. Drug Discov.

[48] Finn J.T., Grunwald M.E., Yau K.W. (1996). Cyclic nucleotide-gated ion channels: an extended family with diverse functions. Annu. Rev. Physiol.

[49] Biel M., Zong X., Ludwig A., Sautter A., Hofmann F. (1999). Structure and function of cyclic nucleotide-gated channels. Rev. Physiol. Biochem. Pharmacol.

[50] Frings S., Seifert R., Godde M., Kaupp U.B. (1995). Profoundly different calcium permeation and blockage determine the specific function of distinct cyclic nucleotide-gated channels. Neuron.

[51] Abi-Gerges A., Richter W., Lefebvre F., Mateo P., Varin A., Heymes C., Samuel J.L., Lugnier C., Conti M., Fischmeister R. (2009). Decreased expression and activity of cAMP phosphodiesterases in cardiac hypertrophy and its impact on beta-adrenergic cAMP signals. Circ. Res.

[52] Ghigo A., Perino A., Mehel H., Zahradnikova A., Morello F., Leroy J., Nikolaev V.O., Damilano F., Cimino J., De Luca E. (2012). Phosphoinositide 3-kinase gamma protects against catecholamine-induced ventricular arrhythmia through protein kinase A-mediated regulation of distinct phosphodiesterases. Circulation.

[53] Rochais F., Vandecasteele G., Lefebvre F., Lugnier C., Lum H., Mazet J.L., Cooper D.M., Fischmeister R. (2004). Negative feedback exerted by cAMP-dependent protein kinase and cAMP phosphodiesterase on subsarcolemmal cAMP signals in intact cardiac myocytes: An *in vivo* study using adenovirus-mediated expression of CNG channels. J. Biol. Chem.

[54] Rich T.C., Fagan K.A., Nakata H., Schaack J., Cooper D.M., Karpen J.W. (2000). Cyclic nucleotide-gated channels colocalize with adenylyl cyclase in regions of restricted cAMP diffusion. J. Gen. Physiol.

[55] Rich T.C., Tse T.E., Rohan J.G., Schaack J., Karpen J.W. (2001). *In vivo* assessment of local phosphodiesterase activity using tailored cyclic nucleotide-gated channels as cAMP sensors. J. Gen. Physiol.

[56] Fesenko E.E., Kolesnikov S.S., Lyubarsky A.L. (1985). Induction by cyclic GMP of cationic conductance in plasma membrane of retinal rod outer segment. Nature.

[57] Koutalos Y., Nakatani K., Yau K.W. (1995). The cGMP-phosphodiesterase and its contribution to sensitivity regulation in retinal rods. J. Gen. Physiol.

[58] Baylor D.A., Lamb T.D., Yau K.W. (1979). Responses of retinal rods to single photons. J. Physiol.

[59] Nakatani K., Yau K.W. (1988). Guanosine 3′,5′-cyclic monophosphate-activated conductance studied in a truncated rod outer segment of the toad. J. Physiol.

[60] Castro L.R., Verde I., Cooper D.M., Fischmeister R. (2006). Cyclic guanosine monophosphate compartmentation in rat cardiac myocytes. Circulation.

[61] Castro L.R., Schittl J., Fischmeister R. (2010). Feedback control through cGMP-dependent protein kinase contributes to differential regulation and compartmentation of cGMP in rat cardiac myocytes. Circ. Res.

[62] Förster T. (1948). Zwischenmolekulare Energiewanderung und Fluoreszenz. Ann. Physik.

[63] Wu P., Brand L. (1994). Resonance energy transfer: methods and applications. Anal. Biochem.

[64] Adams S.R., Harootunian A.T., Buechler Y.J., Taylor S.S., Tsien R.Y. (1991). Fluorescence ratio imaging of cyclic AMP in single cells. Nature.

[65] Gorbunova Y.V., Spitzer N.C. (2002). Dynamic interactions of cyclic AMP transients and spontaneous Ca(2+) spikes. Nature.

[66] Bacskai B.J., Hochner B., Mahaut-Smith M., Adams S.R., Kaang B.K., Kandel E.R., Tsien R.Y. (1993). Spatially resolved dynamics of cAMP and protein kinase A subunits in Aplysia sensory neurons. Science.

[67] Hempel C.M., Vincent P., Adams S.R., Tsien R.Y., Selverston A.I. (1996). Spatio-temporal dynamics of cyclic AMP signals in an intact neural circuitm. Nature.

[68] Webb R.J., Marshall F., Swann K., Carroll J. (2002). Follicle-stimulating hormone induces a gap junction-dependent dynamic change in [cAMP] and protein kinase a in mammalian oocytes. Dev. Biol.

[69] Takeda N., Kyozuka K., Deguchi R. (2006). Increase in intracellular cAMP is a prerequisite signal for initiation of physiological oocyte meiotic maturation in the hydrozoan Cytaeis uchidae. Dev. Biol.

[70] Goaillard J.M., Vincent P.V., Fischmeister R. (2001). Simultaneous measurements of intracellular cAMP and L-type Ca2+ current in single frog ventricular myocytes. J. Physiol.

[71] Tsien R.Y. (1998). The green fluorescent protein. Annu. Rev. Biochem.

[72] Zaccolo M., De Giorgi F., Cho C.Y., Feng L., Knapp T., Negulescu P.A., Taylor S.S., Tsien R.Y., Pozzan T. (2000). A genetically encoded, fluorescent indicator for cyclic AMP in living cells. Nat. Cell. Biol..

[73] Zaccolo M., Pozzan T. (2002). Discrete microdomains with high concentration of cAMP in stimulated rat neonatal cardiac myocytes. Science.

[74] Mongillo M., McSorley T., Evellin S., Sood A., Lissandron V., Terrin A., Huston E., Hannawacker A., Lohse M.J., Pozzan T. (2004). Fluorescence resonance energy transfer-based analysis of cAMP dynamics in live neonatal rat cardiac myocytes reveals distinct functions of compartmentalized phosphodiesterases. Circ. Res.

[75] Lehnart S.E., Wehrens X.H., Reiken S., Warrier S., Belevych A.E., Harvey R.D., Richter W., Jin S.L., Conti M., Marks A.R. (2005). Phosphodiesterase 4D deficiency in the ryanodine-receptor complex promotes heart failure and arrhythmias. Cell.

[76] Warrier S., Belevych A.E., Ruse M., Eckert R.L., Zaccolo M., Pozzan T., Harvey R.D. (2005). Beta-adrenergic- and muscarinic receptor-induced changes in cAMP activity in adult cardiac myocytes detected with FRET-based biosensor. Am. J. Physiol. Cell Physiol.

[77] Diller T.C., Madhusudan, Xuong N.H., Taylor S.S. (2001). Molecular basis for regulatory subunit diversity in cAMP-dependent protein kinase: crystal structure of the type II beta regulatory subunit. Structure.

[78] Nikolaev V.O., Bunemann M., Hein L., Hannawacker A., Lohse M.J. (2004). Novel single chain cAMP sensors for receptor-induced signal propagation. J. Biol. Chem.

[79] Allen M.D., Zhang J. (2006). Subcellular dynamics of protein kinase A activity visualized by FRET-based reporters. Biochem. Biophys Res. Commun.

[80] Zhang J., Hupfeld C.J., Taylor S.S., Olefsky J.M., Tsien R.Y. (2005). Insulin disrupts beta-adrenergic signalling to protein kinase A in adipocytes. Nature.

[81] Zhang J., Ma Y., Taylor S.S., Tsien R.Y. (2001). Genetically encoded reporters of protein kinase A activity reveal impact of substrate tethering. Proc. Natl. Acad. Sci. USA.

[82] Depry C., Allen M.D., Zhang J. (2011). Visualization of PKA activity in plasma membrane microdomains. Mol. Biosyst.

[83] DiPilato L.M., Cheng X., Zhang J. (2004). Fluorescent indicators of cAMP and Epac activation reveal differential dynamics of cAMP signaling within discrete subcellular compartments. Proc. Natl. Acad. Sci. USA.

[84] Ponsioen B., Zhao J., Riedl J., Zwartkruis F., van der Krogt G., Zaccolo M., Moolenaar W.H., Bos J.L., Jalink K. (2004). Detecting cAMP-induced Epac activation by fluorescence resonance energy transfer: Epac as a novel cAMP indicator. EMBO Rep.

[85] Nikolaev V.O., Gambaryan S., Engelhardt S., Walter U., Lohse M.J. (2005). Real-time monitoring of the PDE2 activity of live cells: hormone-stimulated cAMP hydrolysis is faster than hormone-stimulated cAMP synthesis. J. Biol. Chem.

[86] Calebiro D., Nikolaev V.O., Gagliani M.C., de Filippis T., Dees C., Tacchetti C., Persani L., Lohse M.J. (2009). Persistent cAMP-signals triggered by internalized G-protein-coupled receptors. PLoS Biol.

[87] Violin J.D., DiPilato L.M., Yildirim N., Elston T.C., Zhang J., Lefkowitz R.J. (2008). beta2-adrenergic receptor signaling and desensitization elucidated by quantitative modeling of real time cAMP dynamics. J. Biol. Chem.

[88] Nikolaev V.O., Bunemann M., Schmitteckert E., Lohse M.J., Engelhardt S. (2006). Cyclic AMP imaging in adult cardiac myocytes reveals far-reaching beta1-adrenergic but locally confined beta2-adrenergic receptor-mediated signaling. Circ. Res.

[89] Nikolaev V.O., Lohse M.J. (2009). Novel techniques for real-time monitoring of cGMP in living cells. Handb. Exp. Pharmacol..

[90] Honda A., Adams S.R., Sawyer C.L., Lev-Ram V., Tsien R.Y., Dostmann W.R. (2001). Spatiotemporal dynamics of guanosine 3′,5′-cyclic monophosphate revealed by a genetically encoded, fluorescent indicator. Proc. Natl. Acad. Sci. USA.

[91] Russwurm M., Mullershausen F., Friebe A., Jager R., Russwurm C., Koesling D. (2007). Design of fluorescence resonance energy transfer (FRET)-based cGMP indicators: A systematic approach. Biochem. J.

[92] Sato M., Hida N., Ozawa T., Umezawa Y. (2000). Fluorescent indicators for cyclic GMP based on cyclic GMP-dependent protein kinase Ialpha and green fluorescent proteins. Anal. Chem.

[93] Cawley S.M., Sawyer C.L., Brunelle K.F., van der Vliet A., Dostmann W.R. (2007). Nitric oxide-evoked transient kinetics of cyclic GMP in vascular smooth muscle cells. Cell Signal.

[94] Honda A., Moosmeier M.A., Dostmann W.R. (2005). Membrane-permeable cygnets: Rapid cellular internalization of fluorescent cGMP-indicators. Front. Biosci.

[95] Honda A., Sawyer C.L., Cawley S.M., Dostmann W.R. (2005). Cygnets: *In vivo* characterization of novel cGMP indicators and *in vivo* imaging of intracellular cGMP. Methods Mol. Biol.

[96] Mongillo M., Tocchetti C.G., Terrin A., Lissandron V., Cheung Y.F., Dostmann W.R., Pozzan T., Kass D.A., Paolocci N., Houslay M.D. (2006). Compartmentalized phosphodiesterase-2 activity blunts beta-adrenergic cardiac inotropy via an NO/cGMP-dependent pathway. Circ. Res.

[97] Takimoto E., Champion H.C., Belardi D., Moslehi J., Mongillo M., Mergia E., Montrose D.C., Isoda T., Aufiero K., Zaccolo M. (2005). cGMP catabolism by phosphodiesterase 5A regulates cardiac adrenergic stimulation by NOS3-dependent mechanism. Circ. Res.

[98] Nikolaev V.O., Gambaryan S., Lohse M.J. (2006). Fluorescent sensors for rapid monitoring of intracellular cGMP. Nat. Methods.

[99] Niino Y., Hotta K., Oka K. (2009). Simultaneous live cell imaging using dual FRET sensors with a single excitation light. PLoS One.

[100] Iancu R.V., Ramamurthy G., Warrier S., Nikolaev V.O., Lohse M.J., Jones S.W., Harvey R.D. (2008). Cytoplasmic cAMP concentrations in intact cardiac myocytes. Am. J. Physiol. Cell Physiol.

[101] Willemse M., Janssen E., de Lange F., Wieringa B., Fransen J. (2007). ATP and FRET--a cautionary note. Nat. Biotechnol.

[102] Salonikidis P.S., Niebert M., Ullrich T., Bao G., Zeug A., Richter D.W. (2011). An ion-insensitive cAMP biosensor for long term quantitative ratiometric fluorescence resonance energy transfer (FRET) measurements under variable physiological conditions. J. Biol. Chem.

[103] Börner S., Schwede F., Schlipp A., Berisha F., Calebiro D., Lohse M.J., Nikolaev V.O. (2011). FRET measurements of intracellular cAMP concentrations and cAMP analog permeability in intact cells. Nat. Protoc.

[104] Norris R.P., Ratzan W.J., Freudzon M., Mehlmann L.M., Krall J., Movsesian M.A., Wang H., Ke H., Nikolaev V.O., Jaffe L.A. (2009). Cyclic GMP from the surrounding somatic cells regulates cyclic AMP and meiosis in the mouse oocyte. Development.

[105] Piggott L.A., Hassell K.A., Berkova Z., Morris A.P., Silberbach M., Rich T.C. (2006). Natriuretic peptides and nitric oxide stimulate cGMP synthesis in different cellular compartments. J. Gen. Physiol.

[106] Tsai E.J., Kass D.A. (2009). Cyclic GMP signaling in cardiovascular pathophysiology and therapeutics. Pharmacol. Ther.

[107] Baird G.S., Zacharias D.A., Tsien R.Y. (1999). Circular permutation and receptor insertion within green fluorescent proteins. Proc. Natl. Acad. Sci. USA.

[108] Nausch L.W., Ledoux J., Bonev A.D., Nelson M.T., Dostmann W.R. (2008). Differential patterning of cGMP in vascular smooth muscle cells revealed by single GFP-linked biosensors. Proc. Natl. Acad. Sci. USA.

[109] Isner J.C., Maathuis F.J. (2011). Measurement of cellular cGMP in plant cells and tissues using the endogenous fluorescent reporter FlincG. Plant. J.

[110] Isner J.C., Nuhse T., Maathuis F.J. (2012). The cyclic nucleotide cGMP is involved in plant hormone signalling and alters phosphorylation of Arabidopsis thaliana root proteins. J. Exp. Bot.

[111] Wachten S., Masada N., Ayling L.J., Ciruela A., Nikolaev V.O., Lohse M.J., Cooper D.M. (2010). Distinct pools of cAMP centre on different isoforms of adenylyl cyclase in pituitary-derived GH3B6 cells. J. Cell. Sci.

[112] Mohamed T.M., Oceandy D., Zi M., Prehar S., Alatwi N., Wang Y., Shaheen M.A., Abou-Leisa R., Schelcher C., Hegab Z. (2011). Plasma membrane calcium pump (PMCA4)- neuronal nitric-oxide synthase complex regulates cardiac contractility through modulation of a compartmentalized cyclic nucleotide microdomain. J. Biol. Chem.

[113] Boute N., Jockers R., Issad T. (2002). The use of resonance energy transfer in high-throughput screening: BRET versus FRET. Trends Pharmacol. Sci.

[114] Willoughby D., Cooper D.M. (2008). Live-cell imaging of cAMP dynamics. Nat. Methods.

[115] Pfleger K.D., Eidne K.A. (2006). Illuminating insights into protein-protein interactions using bioluminescence resonance energy transfer (BRET). Nat. Methods.

[116] Prinz A., Diskar M., Erlbruch A., Herberg F.W. (2006). Novel, isotype-specific sensors for protein kinase A subunit interaction based on bioluminescence resonance energy transfer (BRET). Cell Signal.

[117] Barak L.S., Salahpour A., Zhang X., Masri B., Sotnikova T.D., Ramsey A.J., Violin J.D., Lefkowitz R.J., Caron M.G., Gainetdinov R.R. (2008). Pharmacological characterization of membrane-expressed human trace amine-associated receptor 1 (TAAR1) by a bioluminescence resonance energy transfer cAMP biosensor. Mol. Pharmacol.

[118] Jiang L.I., Collins J., Davis R., Lin K.M., DeCamp D., Roach T., Hsueh R., Rebres R.A., Ross E.M., Taussig R. (2007). Use of a cAMP BRET sensor to characterize a novel regulation of cAMP by the sphingosine 1-phosphate/G13 pathway. J. Biol. Chem.

[119] Matthiesen K., Nielsen J. (2011). Cyclic AMP control measured in two compartments in HEK293 cells: phosphodiesterase K(M) is more important than phosphodiesterase localization. PLoS One.

[120] Biswas K.H., Sopory S., Visweswariah S.S. (2008). The GAF domain of the cGMP-binding, cGMP-specific phosphodiesterase (PDE5) is a sensor and a sink for cGMP. Biochemistry.

[121] Jager R., Schwede F., Genieser H.G., Koesling D., Russwurm M. (2010). Activation of PDE2 and PDE5 by specific GAF ligands: delayed activation of PDE5. Br. J. Pharmacol.

[122] Hayes J.S., Brunton L.L., Brown J.H., Reese J.B., Mayer S.E. (1979). Hormonally specific expression of cardiac protein kinase activity. Proc. Natl. Acad. Sci. USA.

[123] Jurevicius J., Fischmeister R. (1996). cAMP compartmentation is responsible for a local activation of cardiac Ca2+ channels by beta-adrenergic agonists. Proc. Natl. Acad. Sci. USA.

[124] Rochais F., Abi-Gerges A., Horner K., Lefebvre F., Cooper D.M., Conti M., Fischmeister R., Vandecasteele G. (2006). A specific pattern of phosphodiesterases controls the cAMP signals generated by different Gs-coupled receptors in adult rat ventricular myocytes. Circ. Res.

[125] Terrin A., Di Benedetto G., Pertegato V., Cheung Y.F., Baillie G., Lynch M.J., Elvassore N., Prinz A., Herberg F.W., Houslay M.D. (2006). PGE(1) stimulation of HEK293 cells generates multiple contiguous domains with different [cAMP]: role of compartmentalized phosphodiesterases. J. Cell. Biol.

[126] Di Benedetto G., Zoccarato A., Lissandron V., Terrin A., Li X., Houslay M.D., Baillie G.S., Zaccolo M. (2008). Protein kinase A type I and type II define distinct intracellular signaling compartments. Circ. Res.

[127] Herget S., Lohse M.J., Nikolaev V.O. (2008). Real-time monitoring of phosphodiesterase inhibition in intact cells. Cell Signal.

[128] Sin Y.Y., Edwards H.V., Li X., Day J.P., Christian F., Dunlop A.J., Adams D.R., Zaccolo M., Houslay M.D., Baillie G.S. (2011). Disruption of the cyclic AMP phosphodiesterase-4 (PDE4)-HSP20 complex attenuates the beta-agonist induced hypertrophic response in cardiac myocytes. J. Mol. Cell Cardiol.

[129] Liu S., Zhang J., Xiang Y.K. (2011). FRET-based direct detection of dynamic protein kinase A activity on the sarcoplasmic reticulum in cardiomyocytes. Biochem. Biophys. Res. Commun.

[130] Dyachok O., Isakov Y., Sagetorp J., Tengholm A. (2006). Oscillations of cyclic AMP in hormone-stimulated insulin-secreting beta-cells. Nature.

[131] Sample V., DiPilato L.M., Yang J.H., Ni Q., Saucerman J.J., Zhang J. (2012). Regulation of nuclear PKA revealed by spatiotemporal manipulation of cyclic AMP. Nat. Chem. Biol.

[132] Nikolaev V.O., Moshkov A., Lyon A.R., Miragoli M., Novak P., Paur H., Lohse M.J., Korchev Y.E., Harding S.E., Gorelik J. (2010). Beta2-adrenergic receptor redistribution in heart failure changes cAMP compartmentation. Science.

[133] Steyer J.A., Almers W. (2001). A real-time view of life within 100 nm of the plasma membrane. Nat. Rev. Mol. Cell Biol.

[134] Dyachok O., Idevall-Hagren O., Sagetorp J., Tian G., Wuttke A., Arrieumerlou C., Akusjarvi G., Gylfe E., Tengholm A. (2008). Glucose-induced cyclic AMP oscillations regulate pulsatile insulin secretion. Cell Metab.

